# Treatment Challenges in Severe Eosinophilic Asthma: Differential Response to Anti-IL-5 and Anti-IL-5R Therapy

**DOI:** 10.3390/ijms22083969

**Published:** 2021-04-12

**Authors:** Agamemnon Bakakos, Nikoleta Rovina, Petros Bakakos

**Affiliations:** Department of Respiratory Medicine, National and Kapodistrian University of Athens, 157 72 Athens, Greece; agabak@hotmail.com (A.B.); nikrovina@med.uoa.gr (N.R.)

**Keywords:** asthma, severe eosinophilic asthma, anti-IL-5, anti-IL-5R, mepolizumab, benralizumab

## Abstract

Severe asthma greatly affects patients’ quality of life. Major advances have occurred in the management of severe eosinophilic asthma the past few years due to the new targeted biological therapies. There are three anti-IL-5 mAbs, mepolizumab, reslizumab and benralizumab. Despite the different mechanism of blocking IL-5 the clinical effects are quite similar as randomized controlled trials and real-life studies have shown. Moreover, there are reports of responding to one after failing to respond to another anti-IL-5 therapy. Accordingly, it is challenging to explore the possible differences in the response to anti-IL-5 treatments. This might help us not only understand possible mechanisms that contribute to the resistance to treatment in this particular asthma endotype, but also to phenotype within severe eosinophilic asthma in order to treat our patients more efficiently.

## 1. Introduction

Bronchial asthma is a disease which consists of chronic airway inflammation, structural changes to the bronchial tree and airway hyperresponsiveness (AHR). Its worldwide prevalence is estimated to be around 4.3%, with some countries experiencing a higher burden of the disease, such as the United States of America and Australia, up to 10% [1]. Almost 95 out of 100 asthmatics worldwide experience mild to moderate symptoms, which can be controlled by treatment with inhaled corticosteroids (ICS) and long-acting beta-2 receptor agonists (LABA). However, a small proportion of them need an escalation of treatment with either oral corticosteroids (OCS) or novel biologics targeting specific molecular pathways, which intertwine with the severity of symptoms and are specific to each patient. 

This subgroup is termed “severe asthmatics” and includes individuals whose symptoms cannot be controlled under high dose ICS and LABA treatment, or need OCS for several months each year in order to overcome their symptoms. It should be noted that before characterizing asthma as “severe uncontrolled”, a period of surveillance is needed to ensure that it is indeed properly treated and that the patient complies with the use of his medication [2]. This “hard to treat” asthma urged experts to delve deeper into its molecular pathways and ultimately recognize the need to endotype each patient. Eosinophils were quickly revealed to play a predominant role in the pathogenesis of severe asthma, currently known as T2 high asthma. Knowledge about this specific endotype is rapidly growing along with our arsenal of monoclonal antibodies targeting specific mediators involved in differentiation and activation of eosinophils. Not surprisingly, biologics have already proven their great efficacy; however, there still remains quite a few unanswered questions as we continue to experiment not only with their use but also with the switch from one biological to another. 

## 2. Eosinophils in the Spotlight of T2 High Inflammation

Eosinophils have drawn great interest over the past decade since the breakthrough with the discovery of monoclonal antibodies targeting IL-5 and its receptor, a major cytokine which promotes eosinophil migration to the lungs, as well as their proliferation and survival [3]. Until 2012 the only pathway experts could target in severe asthma was IgE with the use of omalizumab, a monoclonal antibody which inhibits IgE and has already improved the quality of life in patients with a predominant allergic endotype. The importance of eosinophils and the cytokines which affect their behavior in the lungs can be highlighted by the fact that they can be stimulated by multiple molecular pathways and lead to T2 high inflammation [3]. 

The T2 high endotype includes all the cytokines initially believed to be solely observed when CD4 T helper 2 (TH2) cells are stimulated mainly by environmental allergens. These triggers cause an immediate response by these adaptive immune system cells by initiating the production of cytokines like IL-4, IL-5 and IL-13, leading to eosinophil recruitment and activation [4]. Recently, the identification of a previously unknown cellular population in lung tissue brought significant changes to this simplistic view. The Innate Lymphoid Cells 2 (ILC2) were discovered to possess the ability to promote a similar T2 high response leading to lung eosinophilia and airway inflammation. Unlike the previously mentioned TH2 cells that are part of adaptive immunity, ILC2 demonstrate the effects of innate immunity in severe asthma. ILC2 have been shown to secrete IL-5 constitutively and even express IL-13 while greatly enhancing IL-5 secretion in circumstances of type 2 inflammation, leading to the activation of the T2 inflammatory cascade. More specifically, studies have underlined the importance of IL-13 as an activator of eotaxin-1, a chemokine which acts as an eosinophil chemoattractant and binds to the CCR3 receptor on eosinophils in the early stages of the T2 inflammatory process, orchestrating their migration to the lungs synergistically with IL-5 [5]. ILC2 respond to stimuli called alarmins, cytokines produced by epithelial lung cells in situations of bacterial contact or epithelial damage. These are IL-25, IL-33 and Thymic Stromal Lymphopoietin (TSLP). IL-33 has been clearly associated with the activation of both TH2 cells and ILC2, which leads to IL-5 and IL-13 production. Interestingly, injecting IL-33 in T helper deficient mice could still lead to airway eosinophilia, thus further establishing the role of ILC2. Furthermore, blockage of the IL-33 pathway by inhibiting either IL-33 via antibodies or the ST2 receptor which interacts with it, led to a significant reduction of lung infiltration by eosinophils and AHR [6]. Additionally, ILC2 have been implied with responding to additional mediators in mice, such as the leukotriene D4 (LTD4). Unlike the aforementioned alarmins, LTD4 can precipitate in the production of IL-4 from ILC2 cells, pinpointing that there is more to discover concerning the initial activation of these cells [7]. The T2 high inflammatory pathway has been proven to be far more elaborate than originally estimated. The function of ILC2 cells could be crucial in order to target simultaneously more than one cytokine in our effort to treat severe eosinophilic asthma. 

Eosinophils are produced in the bone marrow like all hemopoietic cells. IL-5, along with other cytokines like IL-3 and Granulocyte Macrophage—Colony Stimulating Factor (GM-CSF), is crucial to their survival. This was demonstrated in IL-5-deficient mice, which could not produce an adequate number of eosinophils and evidently could not exhibit a T2 high response. It should be noted that eosinophils continue to express the IL-5 receptor on their membrane even after maturation and migration to the lungs, deeming IL-5 crucial to their survival, which in turn means that blocking IL-5 would not only halt their production but also rapidly lead to eosinophil attenuation in bronchial tissue [8,9,10]. As previously noted, the migration of eosinophils to the lungs is mainly coordinated by IL-5 and eotaxin-1. It is clinically important to underline that eotaxin-1 cannot mobilize eosinophils without synergia with IL-5, meaning that blocking IL-5 is far more crucial treatment-wise, since eotaxin-1 alone can only lead to blood eosinophilia but not to tissue infiltration. On the contrary, blocking eotaxin-1 alone cannot prevent eosinophil migration to the lungs, although it surely dampens the effectiveness of IL-5 [11]. Eotaxin-2 synergizes with IL-5 and drives the production of IL-13, while eotaxin-3 is thought to prolong eosinophil recruitment in the lungs [11].

Airway hyperresponsiveness and remodeling are hallmarks of bronchial asthma. It is no surprise that eosinophils are heavily associated with both processes. Their degranulation in lung tissue can lead to direct damage of the airways through the release of factors such as leukotrienes and Major Basic Protein (MBP). Leukotrienes directly cause severe bronchoconstriction, induce production of histamine and activate basophils and mast cells, thus fueling the flames of inflammation [12]. Nevertheless, abolishing eosinophils in vitro could not result in cessation of AHR, confirming that their role might be important but also limited [13]. On the other hand, eosinophils are involved in airway remodeling. Numerous studies have demonstrated that Matrix Metaloproteinase-9 (MMP-9) can be excreted from eosinophils, leading to the accumulation of inflammation-related cytokines like Tumor Necrosis Factor-a (TNF-a) and IL-1 beta in the lungs. Furthermore, MMP-9 levels are much higher in asthmatics and its levels are steadily raised in patients with persistent eosinophilia and severe airway remodeling [14]. Last but not least, studies in mice which targeted either eosinophils directly through abolishing genes crucial to their production in bone marrow or IL-5 and its receptor through monoclonal antibodies, managed to demonstrate protection against airway remodeling when compared to wild type mice, evident by the significant reduction of extracellular membrane proteins deposited in the lumen of their bronchial tree [15]. 

Summarizing, eosinophils and several cytokines like IL-5, implied in their production, migration and infiltration of the lungs, are key features of inflammation in asthma. The T2 high endotype has multiple molecular pathways with both the allergic and non-allergic cascade revolving around eosinophils. Therefore, targeting them via monoclonal antibodies in an attempt to treat asthmatics suffering from severe asthma was the “next big thing”.

## 3. Randomized Control Trials Targeting IL-5 and IL-5R

### 3.1. Mepolizumab

The first biologic that targeted eosinophils in the setting of T2 high endotype was mepolizumab, a humanized anti-IL-5 monoclonal antibody that blocks the connection between circulating IL-5 and its receptor complex on cells, particularly eosinophils and basophils. One of the largest randomized control trials (RCTs) ever conducted in asthma was published in 2012 under the acronym DREAM. Researchers recruited 621 patients with a validated status of severe asthma refractory to treatment, as determined by a high annual exacerbation rate (≥2 the previous year) requiring oral corticosteroids. The key of their success was that patients were enrolled only with signs of eosinophilic inflammation, such as FeNO >50 ppb, peripheral blood eosinophils >300 × 10^6/^L or sputum eosinophils >3%. All three mepolizumab doses that were tested managed to drastically reduce the annual exacerbation rate of patients in the 52-week surveillance period and almost completely depleted eosinophils from sputum and peripheral blood. However, no improvement in lung function test parameters like FEV_1_ was observed and patients did not report improved control of their symptoms in their Asthma Quality of Life Questionnaire (AQLQ) [16]. Nevertheless, in 2014, a MENSA study was the first to depict the positive effects of mepolizumab treatment in severe asthmatics with eosinophilic predominant endotype on FEV_1_ and asthma control as well. The annual rate of exacerbations in the 576 participants was almost halved during the 52 weeks of its duration [17]. Simultaneously, a SIRIUS study explored the corticosteroid sparing effects of anti-IL-5 treatment by achieving a reduction in oral corticosteroid dose in the subgroup receiving mepolizumab versus placebo. Meanwhile, the rate of exacerbations was drastically reduced despite the lowering of the OCS dose and patients reported improved asthma control and quality of life [18]. Many post-hoc analyses utilized the results of these studies in order to determine secondary endpoints and give experts a better understanding of treatment with an anti-IL-5 agent. A key result was that the higher the number of peripheral blood eosinophils, the more likely the response to mepolizumab was [19]. Finally, its safety was also well validated in follow-up studies, such as the COSMOS study, which was recently finalized reporting no safety concern issues [20]. 

### 3.2. Benralizumab

An interesting twist was the idea of targeting the IL-5 receptor instead of IL-5 itself. This came to fruition with the biologic benralizumab. Its ability to bind to IL-5R and lead both eosinophils and basophils to apoptosis not only by depriving them from IL-5 but also by drawing NK cells, causing a fast and efficient antibody-dependent-cell-mediated-cytotoxicity, quickly depletes their population in both the peripheral blood and the lungs [21]. Two RCTs under the names of SIROCCO and CALIMA tested benralizumab’s efficacy in severe asthmatics with blood eosinophils of at least 300 cells/μL. Their results were similar, reporting a significant reduction in the annual exacerbation rate with a concomitant increase in FEV_1_ and an improvement in symptom control [22,23]. Similar to the initial trials with mepolizumab, the next question was whether benralizumab could sustain the same corticosteroid sparing effects with its predecessor. The ZONDA study clearly demonstrated that treatment with benralizumab could indeed reduce the OCS burden, since after a 28-week period during which patients were randomized to either anti-IL-5R treatment or placebo, half of them managed to completely stop OCS without worsening their asthma control [24]. Its safety was also well established in a 2-year follow-up study [25].

### 3.3. Reslizumab

The last child of the anti-IL-5/anti-IL-5R family is reslizumab. Similar to mepolizumab it targets IL-5 directly; however, it has several structural and dosing differences that cause it to shine in specific settings. It is injected intravenously instead of the subcutaneous way that is used for the other available mAbs and its dose is weight-adjusted. Two phase III studies were run after recruiting 953 patients with severe eosinophilic asthma and randomized them to either reslizumab or placebo. Both managed to show a decline in patients’ annual exacerbation rate and improvement of their lung function and quality of life [26]. Although reslizumab does not savor the glory of its counterparts, the RCT findings encouraged experts to test it in clinical practice due to its weight-dependent dose, a unique feature that makes it a suitable candidate for non-responders to other anti-IL-5 agents. 

## 4. Real-Life Studies Targeting IL-5 and IL-5R

### 4.1. Mepolizumab

Treatment with mepolizumab has been proven to effectively reduce the significant and long-standing disease burden in patients with severe eosinophilic asthma in the real-life setting. In a real-world retrospective observational longitudinal study including 78 patients with severe eosinophilic asthma, mepolizumab was considered beneficial and was therefore continued in 75.6% of patients 1 year after the initiation of treatment. Severe asthma exacerbations per year were significantly reduced after 12 months of treatment accompanied by a reduction of 0.80 points in ACQ, and an increase in FEV_1_ compared to baseline. A reduction in OCS usage was also observed (51.3% at baseline vs. 15.4%), 12 months after drug initiation and importantly, no serious adverse events related to mepolizumab treatment were reported [27]. Similar findings come from a retrospective study of 138 patients treated with mepolizumab for at least 12 months in Italy where mepolizumab confirmed its effectiveness in significantly reducing exacerbation rates and OCS usage, thus replicating the efficacy and safety profile shown in RCTs [28].

In line with the previous results, a real-life study from Belgium including 116 patients with severe eosinophilic asthma followed up for at least 18 months assessed the rate of exacerbations, OCS usage, FeNO, lung function, asthma control and quality of life at baseline, after 6 months and then every year. Importantly, sputum induction and a peripheral blood count of eosinophils were performed at the same time-points. Sputum eosinophil counts were reduced by 60% after 6 months of treatment while blood eosinophil counts were reduced by 98%. A significant reduction in exacerbation rate by 85% was observed after 6 months, which was maintained over time. A significant and preserved reduction by 50% in the dose of OCS was also recorded. Patients significantly improved their asthma control, as assessed by ACT and ACQ scores as well as quality of life (AQLQ score) at 6 months and this was also maintained throughout the follow-up. Eventually, a progressive improvement in FEV_1_ that reached significance after 18 months was observed, and this was more pronounced in patients with a higher baseline sputum eosinophil percentage [29].

The long-term efficacy and safety of mepolizumab was also shown in the retrospective, observational study from France, demonstrating significant improvements in reducing exacerbations and OCS use over a period of 24 months [30].

A real-life study of a larger sample size comes from the Australian mepolizumab Registry (AMR) including 309 patients. These patients had poor asthma control, frequent exacerbations and almost half of them (47%) required daily OCS. Most of them had comorbidities, while the median baseline peripheral blood eosinophil count was 590 cells/μL. Mepolizumab reduced significantly exacerbations requiring OCS compared to the previous year as well as hospitalizations. A global improvement in asthma control, in quality of life and in lung function was also observed. Higher blood eosinophils and the later age of asthma onset were predictors of a better response, while predictors of a lesser response were the male sex and BMI ≥30. A subgroup of super-responders presented a more intense T2 high profile and fewer comorbidities [31].

Another study from the UK including 99 patients with severe eosinophilic asthma receiving mepolizumab determined the prognostic factors associated with response and super-response to treatment. In this study, response was defined as ≥50% reduction in exacerbations, or for patients requiring maintenance oral corticosteroids, ≥50% reduction in prednisolone dose. Super-responders were defined as exacerbation-free and off maintenance OCS at 1 year of treatment. Nasal polyposis, a lower BMI and a lower maintenance OCS requirement at baseline were associated with better outcomes. Twelve-month response was identifiable in over 90% of patients by week 24 of treatment [32].

### 4.2. Benralizumab

One of the first real-life studies assessing the therapeutic effect of benralizumab in severe eosinophilic asthma included 13 patients who were evaluated at baseline and 4 weeks after drug administration. A rapid drop in blood eosinophil count (from 814.7 ± 292.3 cells/μL at baseline to 51.3 ± 97.5 cells/μL) was already noted 4 weeks after the first injection and this was associated with clinically significant improvements in ACT and FEV_1_, allowing the complete cessation of OCS [33].

Renner et al. showed that within a real life setting, benralizumab exerts quite a rapid and effective improvement in particular outcomes from the first 24 h of administration in patients with severe eosinophilic asthma. They recorded the response of 56 patients that were included in the study right after the first dosing, in 24 h and one week after treatment initiation and thereafter at each administration for up to 48 weeks. The median ACT score improvement was 7.0 points at 4 weeks and the median ACQ6 score improved by 1.17 points after one week. Furthermore, median FEV_1_ significantly increased within the first 24 h, with further improvements after one week of administration. These improvements remained statistically and clinically significant until the end of the study. Although an immediate depletion of the peripheral blood eosinophil count was observed, there was no apparent trend in FeNO measurement despite FeNO being significantly reduced compared to baseline at specific timepoints (week 8, 16, 32 and 40). This study showed no differences in the effect of improving asthma control between non-smokers and former smokers and also between asthmatics with and without reversibility. The OCS sparing effect of benralizumab was not evaluated in this study because only 12 patients received OCS at baseline [34]. 

By suppressing eosinophilic inflammation, benralizumab not only decreases asthma exacerbations but at the same time improved airflow limitation and lung hyperinflation. This was shown in a study that enrolled 22 allergic patients with severe eosinophilic asthma. Benralizumab completely depleted peripheral eosinophils (from 810 to 0 cells/μL), with a concomitant significant decrease in both asthma exacerbation rate (from 4 to 0 exacerbations) and residual volume (from 2720 to 2300 mL) at 24 weeks of treatment [35].

The efficacy and safety of benralizumab was assessed in a real-world cross-sectional multi-center study of a cohort of 42 consecutive patients with severe refractory eosinophilic asthma who received benralizumab for at least 6 months. This study confirmed once again the findings of previous pivotal studies showing a rapid initial improvement in asthma control and lung function, along with a reduction in the number of emergency department visits and the use of inhaled and oral corticosteroids. This study also showed that patients may continue to improve during the first 6 months of treatment, and interestingly, to a greater degree than in pivotal RCT studies. Benralizumab was safe and well-tolerated in the real-life setting [36].

### 4.3. Reslizumab

In a retrospective study, 215 patients were treated with reslizumab for at least ≥ 7 months. Reslizumab was associated with a significant reduction in the proportion of patients experiencing an exacerbation (from 86.0% to 40.5%) as well as in the number of exacerbations per patient (mean 2.84 vs. 0.94). Moreover, significant improvements were observed in FEV_1_ and in ACT score after treatment initiation. Reslizumab showed an oral steroid sparing effect, since more than half of the asthmatics that were receiving maintenance OCS at baseline discontinued them by approximately 10 months after reslizumab initiation [37].

In another real-world study from Ireland including 26 patients, reslizumab was well-tolerated and was associated with a reduction of 79% in the exacerbation rate/year and improvement of ACQ-6 (3.5 to 1.7) at 1 year; 54% of patients were on OCS and 35.7% of them managed to discontinue them after 1 year of treatment [38].

## 5. Differential Response to Anti-IL5s: Observations and Possible Explanations

Mepolizumab and reslizumab act by blocking circulating IL-5 and thus preventing its adhesion to the IL-5R receptor on effector cells, mainly eosinophils. On the other hand, benralizumab interacts with the α subunit of the IL-5R on the surface of eosinophils, basophils and ILC2 cells, thus preventing IL-5 from binding to its receptor. Moreover, the ligation of the Fc constant region of benralizumab to the FcγRIIIa receptor of NK cells leads to antibody-dependent cell cytotoxicity (ADCC), resulting in eosinophil apoptosis [39,40,41].

Despite the differences in the mechanism of action and the intensity of eosinophil depletion, no differences were observed in the RCTs of mepolizumab and benralizumab regarding either the reduction of exacerbations or the corticosteroid sparing effect. The same applies to the real-world studies that have shown even better results compared to RCTs for both monoclonal antibodies (Table 1).

There are no head-to-head studies to compare the two monoclonal antibodies—mepolizumab and benralizumab—and the administration of one or another in a naïve patient with severe eosinophilic asthma is pending on the clinician. Accordingly, the question arises as to whether it is reasonable to administrate the one in case no desirable effect has been observed with the other [21]. 

Weight-adjusted intravenous reslizumab was more effective in reducing sputum eosinophilia compared to fixed-dose SC mepolizumab administered for at least one year with an inadequate response in a study including 10 patients with oral corticosteroid-dependent asthma [42]. Moreover, in a real-world study from the UK including 99 patients, it was shown that a higher BMI was associated with a blunted response to mepolizumab [32]. This indicates that the dose of 100 mg of SC mepolizumab might be insufficient to reduce airway eosinophilia adequately in subjects with a high BMI. In accordance with the above, the DREAM study demonstrated an imminent reduction in blood eosinophils with all three doses administered intravenously (75, 250, 750 mg) but a dose-dependent reduction in sputum eosinophils with the lower dose of 75 mg achieved the lowest reduction [16]. A study evaluating the effect of benralizumab on eosinophil counts in various compartments of patients with eosinophilic asthma demonstrated a 96% reduction in eosinophils of airway mucosal/submucosal biopsies, an 89.9% reduction in sputum eosinophils and complete suppression (100% reduction) in bone marrow, and peripheral blood [43]. In an exploratory analysis of the ZONDA study, median sputum eosinophils decreased from 4.9% to 0.15% after 28 weeks of benralizumab treatment [24]. In a study including steroid-naïve patients with mild atopic asthma, mepolizumab produced a 55% decrease in airway mucosal eosinophils [10], while in another study into severe refractory eosinophilic asthma, the respective reduction of subepithelial airway eosinophils with IV mepolizumab was 48% versus placebo [44].

Peripheral blood eosinophils are used as the main biomarker to identify severe asthmatics eligible for anti-IL-5 or anti-IL-5R treatment. However, it is important to note two things. First, peripheral blood eosinophils have a very limited role in monitoring the response to treatment and second, peripheral blood eosinophils do not always reflect airway eosinophilia. This has been shown in a pediatric population, but also in adults [45]. Of 129 adult asthmatic patients with baseline blood eosinophilia treated with either mepolizumab or reslizumab for at least 4 months, almost 78% of suboptimal responders had raised sputum eosinophils ≥3%, while only 9% presented blood eosinophils ≥400/µL [46]. It seems that the correlation between blood and sputum eosinophils is more prominent in those not receiving OCS and is also more evident as the number of blood eosinophils gets higher [47].

Two major types of eosinophils have been described in the airways. The most known are the eosinophils that emerge from the bone marrow and then are directly recruited to sites of inflammation (inflammatory eosinophils—iEos). A distinct type of eosinophils resides in tissues in homeostatic conditions and present different characteristics. These are called “homeostatic or resident eosinophils” (hEos) [48]. hEos are different from the inflammatory eosinophils (iEos) and have been mostly examined in mice. Both eosinophil populations arise in the bone marrow from the CD34+ progenitor stem cells and specialized CD34+ IL-5R+ hematopoietic progenitor cells. A complex activation of transcription factors plays a role in the eosinophil production, the most important being GATA-1, PU.1, and CAAT enhancer-binding proteins α and ε [49]. Notably, GATA-1 and PU.1 act synergistically for eosinophil production but when it comes to the differentiation of other hematopoietic cells, they are antagonistic [50].

A key difference between the two eosinophil populations is that hEos are differentiated in the bone marrow semi-independently from IL-5. In contrast, iEos need IL-5 in order to be produced from their precursor cells and tracked into the airways [51]. Accordingly, in IL-5 KO mice, the number of hEos in the lung was only reduced by half, clearly showing that these cells could be recruited via different pathways. However, the lack of IL-5 prevented a T2-high response and the recruitment of iEos in the airways. This may also partly explain the presence of eosinophils in asthmatic airways despite treatment with an anti-IL-5 agent [10]. Moreover, mice lacking GM-CSF/IL-3/IL-5 functions did not completely abolish eosinophil production; instead, they had the ability to produce low numbers of eosinophils, indicating that there are more unidentified factors taking part in their development [52]. Another interesting observation is that hEos express several genes not found in the normal iEos that take part in the immunoregulation of the lung and seem to reduce the T2 response after contact with allergens. In this way, not only do they not contribute to the allergic inflammatory process, but they also seem to downregulate the T2 high response. This may be facilitated by the inhibition of dendritic cells [51].

A differential effect of anti-IL-5s and anti-IL-5Rs on these eosinophil populations might explain the differences in their ability to reduce exacerbations and/or the dose of OCS. The individual determination of the numbers of hEos and iEos might be helpful to identify eligible patients for treatment with different biologicals.

Another important process is in situ eosinophilopoiesis in the airways that can occur in severe asthmatics. It was shown that anti-IL-5 treatment with mepolizumab did not abolish eosinophil progenitor cells and did not suppress local airway eosinophil differentiation to mature cells. It has also been demonstrated in the same study that compared to mild asthmatics, the number of CD34+ and CD34+ IL-5R+ hematopoietic progenitor cells was higher in the sputum of severe asthmatics [53]. These data support in situ eosinophilopoiesis as a possible mechanism of persistent airway eosinophilia in severe asthma. It might also explain the discordance between blood and sputum eosinophils in suboptimal responders to mepolizumab or reslizumab who demonstrated persistent sputum eosinophils (>3%) while blood eosinophils were less than 0.4 cells × 10^9^/L [46]. 

Moreover, several cytokines produced by bronchial epithelial cells after a stimulus trigger, such as IL-25, IL-33 and TSLP, known as alarmins, promote the recruitment of eosinophil progenitor cells and trigger ILC2 cells to produce IL-4, IL-5, and IL-13 [54]. In this way, they enhance the homing and in situ maturation of eosinophil progenitor cells in the airways, perpetuating lung eosinophilia [55].

It sounds reasonable to hypothesize that the anti-IL-5s and anti-IL5Rs have differential effects on in situ eosinophilopoiesis and the aim to study such an effect is logical. 

Airway autoimmune phenomena have also been implicated in the suboptimal response to anti-IL-5 mainly in OCS dependent patients. A possible explanation for this is the activation of complement via mepolizumab that is an IgG1-antibody, while reslizumab or benralizumab, which are both IgG4 antibodies, cannot activate C1q [46]. 

Switching from omalizumab to mepolizumab or benralizumab can be performed without a wash-out period and with a favorable effect for patients with the “overlap” phenotype, and thus patients who are eligible for either anti-IgE or anti-IL-5 treatment [21].

Mepolizumab and benralizumab have both been proved effective in reducing exacerbations in severe asthmatics with the «overlap» endotype. In a post-hoc analysis, mepolizumab was effective in reducing exacerbations but also in improving lung function and asthma control regardless of IgE levels and atopic status [56]. Likewise, benralizumab decreased exacerbations and increased FEV_1_ regardless of atopic status and high or low IgE levels (defined as > or <150 kU/L) [57].

These findings were confirmed in real-life studies. In a real-world study including 116 patients from Belgium, the best predictor of a reduction in exacerbations with mepolizumab was a higher baseline total serum IgE level. This implies IgE-mediated mechanisms in propagating IL-5 [29]. In accordance, benralizumab might also interfere with IgE-dependent immune responses, since in a real-world study from Italy all 22 patients recruited who decreased significantly the rate of exacerbations were allergic [35]. Benralizumab causes apoptosis of basophils as well through ADCC [58,59], and as such it reduces the production of IL-4, which is a key cytokine for Th2 cell differentiation and IgE production [60,61]. In a similar manner, benralizumab affects the viability of ILC2, which are a source of IL-4, IL-5, and IL-13 [62,63].

There are very few studies evaluating the response of severe eosinophilic asthma after switching from mepolizumab to benralizumab. In one retrospective real-life study, switching from anti-IL-5 to anti-IL-5Rα therapy due to inadequate treatment response (lack of reduction in exacerbation rate or OCS dosage), persistent impairment of lung function tests or adverse effects, was associated with significantly improved FEV_1_, asthma control and OCS reduction [64]. In another study, 22 patients with severe eosinophilic asthma who had received mepolizumab for ≥24 weeks and presented a suboptimal response defined as either a <50% reduction in OCS dose or a <50% reduction in annualized exacerbation rate, or had an ongoing requirement for ≥7.5 mg prednisolone/day were switched to benralizumab and completed at least 48 weeks of treatment. Exacerbation frequency and OCS dose were significantly reduced while 10 out of 20 patients were able to discontinue OCS [65]. 

## 6. Conclusions

Targeted therapies directed against IL-5 and IL-5R for severe eosinophilic asthma have proved effective mainly in reducing asthma exacerbations and the use of daily oral corticosteroids but also in improving asthma control and lung function. The era of biologics in severe asthma is here to stay and in the current years it has made us very efficient in the treatment of such patients. Despite their comparable effects, anti-IL-5 and anti-IL-5Rs are not the same (Table 2). They have a different mechanism of action and possibly varying effects within the endotype of severe eosinophilic asthma, as shown from the switch from one to another. Distinct eosinophil populations with differential immune functions, in situ eosinophilopoiesis and an effect on airway mucosal eosinophils and basophils may partly explain these differences. The use of biologics in severe asthma has also provided us with the opportunity for a better insight into asthma pathophysiology and its mechanisms. As we gain more experience with biologics, it seems that we would greatly benefit from evaluating those severe asthmatics that have not responded to them or have responded after a switch from one to another. This will explain why phenotypically similar patients respond differently to a treatment modality and would definitely render us wiser in the management of severe asthma.

## Figures and Tables

**Table 1:** Αnti-IL-5/IL-5R monoclonal antibodies effects. Abbreviations used: OCS: oral corticosteroids, ACT: asthma control test, ACQ: asthma control questionnaire.

Reference	mAb	Reduction in Exacerbation Rate	Reduction in OCS	Lung Function Improvement	Asthma Control Improvement (ACT and/or ACQ)
[16] RCT	Mepolizumab	48%	Not studied	−	−
[17] RCT	Mepolizumab	53%	Not studied	+	+
[18] RCT	Mepolizumab	32%	14%	−	+
[22] RCT	Benralizumab	51%	Not studied	+	+
[23] RCT	Benralizumab	28%	Not studied	+	+
[24] RCT	Benralizumab	70%	50%	−	+
[26] RCT	Reslizumab	50–59%	Not studied	+	+
[27] Real life	Mepolizumab	70%	35.9%	+	+
[28] Real life	Mepolizumab	82%	52%		
[29] Real life	Mepolizumab	85%	50%	+	+
[30] Real life	Mepolizumab	86.2%	63%	+	+
[31] Real life	Mepolizumab	66%	36%	+	+
[32] Real life	Mepolizumab	54%	57%	−	+
[34] Real life	Benralizumab	Not studied	Not studied	+	+
[35] Real life	Benralizumab	>90%	82%	+	+
[36] Real life	Benralizumab	85% (ED visits)	50%	+	+
[37]	Reslizumab	67%	53.2%	+	+
[38]	Reslizumab	88%	35.7%	+	+

**Table 2:** Head-to-head anti-IL-5 monoclonal antibody properties comparison. Abbreviations used: SC: subcutaneous, IV: intravenous, mAbs: monoclonal antibodies, OCS: oral corticosteroids, ADCC: antibody-dependent cellular cytotoxicity, BMI: body mass index, (+, ++, +++) denote incremental strength of effect.

	Mepolizumab	Benralizumab	Reslizumab
Dosage	100 mg SC Fixed Dose Q4 Weeks	30 mg SC Fixed Dose Q4 Weeks First 3 Doses Q8 Weeks Following	3–4 mg/kg IV Q4 Weeks
mAb	IgG1	IgG4	IgG4
ADCC	−	+	−
Eosinophil depletion in peripheral blood	+++	+++	+++
Eosinophil depletion in sputm/lungs	++	+++	+++
Reduction of OCS Dependency	+++	+++	+++
Treatment Response Predictors	High blood eosinophils. Late onset. Low OCS burden. Nasal polyps. Lower BMI.	High blood eosinophils. Late onset. Low OCS burden. Nasal polyps. Lower FVC.	High blood eosinophils. Late onset.
Safety	+++	+++	+++
